# Alterations of auditory-evoked gamma oscillations are more pronounced than alterations of spontaneous power of gamma oscillation in early stages of schizophrenia

**DOI:** 10.1038/s41398-023-02511-5

**Published:** 2023-06-27

**Authors:** Mariko Tada, Kenji Kirihara, Daisuke Koshiyama, Tatsuya Nagai, Mao Fujiouka, Kaori Usui, Yoshihiro Satomura, Shinsuke Koike, Kingo Sawada, Jun Matsuoka, Kentaro Morita, Tsuyoshi Araki, Kiyoto Kasai

**Affiliations:** 1 Department of Neuropsychiatry, Graduate School of Medicine, The University of Tokyo, 7-3-1 Hongo, Bunkyo-ku, Tokyo, 113-8655 Japan; 2 International Research Center for Neurointelligence (WPI-IRCN), UTIAS, The University of Tokyo, 7-3-1 Hongo, Bunkyo-ku, Tokyo, 113-0033 Japan; 3 Office for Mental Health Support, Center for Research on Counseling and Support Services, The University of Tokyo, 7-3-1 Hongo, Bunkyo-ku, Tokyo, 113-0033 Japan; 4 The University of Tokyo Institute for Diversity and Adaptation of Human Mind (UTIDAHM), 3-8-1 Komaba, Meguro-ku, Tokyo, 153-8902 Japan; 5 Center for Evolutionary Cognitive Sciences, Graduate School of Arts and Sciences, The University of Tokyo, 3-8-1 Komaba, Meguro-ku, Tokyo, 153-8902 Japan

**Keywords:** Schizophrenia, Physiology, Neuroscience

## Abstract

Several animal models of schizophrenia and patients with chronic schizophrenia have shown increased spontaneous power of gamma oscillations. However, the most robust alterations of gamma oscillations in patients with schizophrenia are reduced auditory–oscillatory responses. We hypothesized that patients with early-stage schizophrenia would have increased spontaneous power of gamma oscillations and reduced auditory–oscillatory responses. This study included 77 participants, including 27 ultra-high-risk (UHR) individuals, 19 patients with recent-onset schizophrenia (ROS), and 31 healthy controls (HCs). The auditory steady-state response (ASSR) and spontaneous power of gamma oscillations measured as induced power during the ASSR period were calculated using electroencephalography during 40-Hz auditory click-trains. The ASSRs were lower in the UHR and ROS groups than in the HC group, whereas the spontaneous power of gamma oscillations in the UHR and ROS groups did not significantly differ from power in the HC group. Both early-latency (0–100 ms) and late-latency (300–400 ms) ASSRs were significantly reduced and negatively correlated with the spontaneous power of gamma oscillations in the ROS group. In contrast, UHR individuals exhibited reduced late-latency ASSR and a correlation between the unchanged early-latency ASSR and the spontaneous power of gamma oscillations. ASSR was positively correlated with the hallucinatory behavior score in the ROS group. Correlation patterns between the ASSR and spontaneous power of gamma oscillations differed between the UHR and ROS groups, suggesting that the neural dynamics involved in non-stimulus-locked/task modulation change with disease progression and may be disrupted after psychosis onset.

## Introduction

Altered gamma oscillations are a key feature of brain dysfunction in schizophrenia [1, 2]; they reflect neural dynamics, the information processing substrates of the brain. Gamma oscillations are useful for translational research because they can be recorded at multiple levels, from single-unit recordings in animals to scalp electroencephalograms (EEGs) in humans [3].

Previous studies have revealed altered gamma oscillations in patients with schizophrenia. Reduced gamma-band auditory steady-state response (ASSR) is the most robust alteration [4] in chronic [5, 6] and first-episode [7, 8] psychosis. Multiple animal studies have explored the mechanisms underlying ASSR alterations in various diseases [9]. In a recent study, we found ASSR deficits in pre-onset ultra-high-risk (UHR) individuals [10, 11], indicating reduced gamma oscillations before psychosis onset, which may aid early detection and interventions.

The spontaneous power of gamma oscillations and gamma-band ASSR have attracted significant attention. In particular, the role of rest/pre-stimulus-task modulation in the pathophysiology of schizophrenia has been evaluated [12]. Gamma-band ASSR is an EEG response in the gamma-band frequency that is entrained to auditory stimuli, whereas the spontaneous power of gamma oscillations represents gamma-band EEG activity that is not entrained to auditory stimuli. Thus, the spontaneous power of gamma oscillation is non-stimulus-locked, broadband gamma-band activity. A recent study showed that, in contrast to gamma-band ASSR, spontaneous (pre-stimulus/non-stimulus-locked) gamma power was increased in patients with chronic schizophrenia; the increase was inversely related to the reduction in gamma-band ASSR [13, 14]. Northoff and Gomez-Pilar [12] found that abnormal cognition and positive symptoms in schizophrenia are related to other abnormalities (termed “state dependence”), rather than an increase or decrease in pre-stimulus or task-related activities. Therefore, it is important to explore both task-related activities (gamma-band ASSR) and non-stimulus-locked spontaneous gamma power in patients with schizophrenia.

Multiple animal studies have shown that reduced NMDA receptor signaling causes increased spontaneous power of gamma oscillations [15–18] and reduced gamma-band ASSR [19, 20]. ASSR develops during adolescence [21–24] because of the maturation of parvalbumin-positive ɣ-aminobutyric acid (GABA)-ergic interneuron function, which is altered in patients with schizophrenia [25]. Previous studies of UHR individuals [26, 27], patients with first-episode psychosis [28], and animal models [27] have shown that psychosis onset is related to dysfunction of the N-methyl-d-aspartate (NMDA) receptor and GABAergic interneurons in hippocampal and prefrontal cortices.

The prognosis of schizophrenia can be improved by early detection and intervention [29]. Therefore, UHR individuals and patients with early-stage schizophrenia should be included in the psychosis progression model. To our knowledge, spontaneous power of gamma oscillations and its correlation with gamma-band ASSR have not been investigated in early-stage schizophrenia. We hypothesized that the spontaneous power of gamma oscillations is increased and is associated with gamma-band ASSRs in UHR individuals and patients with recent-onset schizophrenia (ROS); moreover, spontaneous gamma power and gamma-band ASSRs are associated with the hallucinatory behavior score, as indicated by previous studies of patients with chronic schizophrenia [13, 14].

## Materials and methods

### Participants

This study enrolled 77 participants, including 27 UHR individuals (11 women), 19 patients with ROS (8 women), and 31 healthy controls (HCs; 16 women). Clinical assessments and EEG were performed between October 2010 and January 2014 for the UHR group, between December 2010 and August 2014 for the ROS group, and between November 2010 and March 2014 for the HC group. A subset of these participants (14 UHR individuals, 11 patients with ROS, and 21 HCs) were included in our previous study [10]. The Ethical Committee of the University of Tokyo Hospital approved the study protocol (approval no.: 629, 2226). The study procedures were explained to the participants and written informed consent was obtained.

The participants were aged 15–32 years (Table 1). UHR individuals and patients with ROS were recruited from the University of Tokyo Hospital. Details of the recruitment method were reported in our previous Integrated Neuroimaging Studies in Schizophrenia Targeted for Early Intervention and Prevention (IN-STEP) project [30].

**Table 1:** Demographics and clinical characteristics.

	HC	UHR	ROS	Statistics
*N* (men/women)^a^	31 (15/16)	27 (16/11)	19 (11/8)	*χ*^2^ = 0.80, *P* = 0.67
Mean age (years)^b^	22.0 (2.8)	20.9 (3.8)	22.8 (3.6)	*F*(2,74) = 1.85, *P* = 0.17
Premorbid IQ^b^	110.1 (6.8)	105.7 (8.6)	106.7 (9.3)	*F*(2,74) = 3.04, *P* = 0.05
PANSS total^c^			67.4 (24.9)	
Positive			15.2 (6.1)	
Negative			18.7 (7.7)	
General			33.6 (13.1)	
Antipsychotic dose^d^		162.1 (265.3)	400.8 (378.4)	*t*(44) = -2.52, *P* = 0.02
Benzodiazepine dose^e^		5.5 (9.2)	4.6 (7.3)	*t*(44) = 0.33, *P* = 0.74
DOI (days)			448.7 (431.7)	

*DOI* duration of psychotic illness, *HC* healthy control, *IQ* intelligence quotient, *PANSS* Positive and Negative Syndrome Scale, *ROS* recent-onset schizophrenia, *UHR* ultra-high-risk.

Numbers indicate mean (standard deviation) or number of participants.

^a^Chi-square test.

^b^One-way ANOVA; otherwise, *t* tests were used.

^c^The PANSS score of one participant with ROS was not evaluated.

^d^Chlorpromazine.

^e^Diazepam equivalent doses were calculated (mg/day).

Multiple criteria can be used to identify individuals at high risk of psychosis [31, 32]. The Structured Interview for Prodromal Symptoms (SIPS) criteria [33, 34] was used to select UHR individuals. SIPS evaluates attenuated psychotic symptoms, brief intermittent psychotic symptoms, and genetic risk and deterioration.

Patients were included in the ROS group if they had been diagnosed with schizophrenia based on the Diagnostic and Statistical Manual of Mental Disorders, Fourth Edition (DSM-IV), were aged 15–40 years, and had continuous psychotic symptoms for the past 60 months. Most participants with ROS (*n* = 15/19) had first-episode schizophrenia [11], defined as continuous psychotic symptoms within the past 24 months. Patients who had undergone electroconvulsive therapy were excluded. The symptoms of UHR individuals and patients with ROS were evaluated using the Japanese version of the Positive and Negative Syndrome Scale (PANSS) [35–37] and the Global Assessment of Functioning [38, 39].

The Japanese version of the Mini-International Neuropsychiatric Interview 5.0.0 [40, 41] was used to exclude psychiatric disorders in HCs. Individuals with a history of psychiatric illness or first-degree relatives with axis I disorders revealed on an interview with the psychiatrist were excluded.

We excluded participants who had neurological illness, traumatic brain injury with cognitive sequelae or loss of consciousness for >5 min, premorbid intelligence quotient (IQ) < 70 on the Japanese version of the National Adult Reading Test [42, 43], a history of alcohol abuse, or history of substance use. All participants underwent audiometry to detect 1000 Hz tones at 30 dB.

Table 1 presents the clinical characteristics of the study participants. There were no differences in sex ratio or age among the three groups. Although the mean premorbid IQ was higher for the HC group than for the other groups, there was no significant correlation between IQ and gamma oscillations (spontaneous power of gamma oscillations: *P* = 0.38; early-latency intertrial coherence [ITC]: *P* = 0.69; late-latency ITC: *P* = 0.23). Antipsychotic and benzodiazepine doses were converted to chlorpromazine and diazepam equivalent doses, respectively [44]. Antipsychotics and benzodiazepines were used by 15 and 14 participants in the UHR group, respectively, and by 18 and 10 participants in the ROS group, respectively.

### Auditory stimuli and experimental procedures

ASSR is entrained to the frequency and phase of auditory stimuli [45, 46]. The ASSR paradigm has been used in previous studies [6]. Participants were seated in an electrically shielded room and asked to rest with their eyes open. Arousal status was evaluated from a monitoring room adjacent to the measurement room using pre- and post-measurement scores of the Japanese version of the Stanford Sleepiness Scale [47, 48]. The auditory stimuli were click sounds (80 dB SPL, 1 ms) presented in 500-ms trains at 40 Hz. In total, 200 trains were presented in a single block with an intertrain interval of 500 ms. Auditory stimuli were presented binaurally via earphones inserted into the ears (Multi Trigger System; Medical Try System, Tokyo, Japan).

### EEG recording and processing

A 64-channel Geodesic Sensor Net (Electrical Geodesics Inc., Eugene, OR, USA) was used for EEG recording. Methods used for recording and pre-processing analysis have been reported elsewhere [10]. In particular, a high-pass filter (1 Hz) and a notch filter (50 Hz) were applied to the EEG data to remove artifacts. Offline analyses were performed using EEGLAB (http://sccn.ucsd.edu/eeglab) [49]. Independent component analysis was used to remove eye blink, saccadic spike potential, and electromyographic artifacts. Epochs containing muscle activities and body movements were rejected using manual inspection. Artifact-free single epochs were re-referenced to average activity. There was no significant difference in post-processing epoch numbers among the groups (one-way analysis of variance [ANOVA], *F*[2,74] = 1.53, *P* = 0.22; mean number of epochs, HC: 178; UHR: 176; ROS: 183).

Time-frequency analyses with short-term Fourier transformation were performed and the ITC was calculated at the frontocentral electrode (FCz) site, as previously described [10]. The frequency range for ASSR analyses was 36–45 Hz. ITC describes phase consistency across trials, a parameter adopted in multiple previous studies of ASSR [6, 50]. We found no significant differences in the harmonic response (i.e., 80 Hz) of 40-Hz ASSR among groups (one-way ANOVA, *F*[2,74] = 0.34, *P* = 0.71). Therefore, we focused on 40-Hz ASSR. Previously, we found that early-latency (0–100 ms) and late-latency (300–400 ms) ASSRs have distinct characteristics between UHR individuals and patients with first-episode schizophrenia [10]. Moreover, frequency tuning characteristics differed between early- and late-latency ASSRs on subcortical electroencephalography [51], suggesting that they arise from different neurophysiological mechanisms [52]. In particular, Ross and colleagues [52] compared ASSRs with transient auditory responses; they found that, for the 40-Hz ASSR, the first 100 ms is dominated by transient auditory responses, whereas the subsequent response gradually increases until 200 ms and is dominated by a steady-state response, which may be a synchronized oscillation to tone that gradually increases with time. Therefore, we calculated the mean ITC for each time block (early: 0–100 ms; late: 300–400 ms), as in our previous study.

Regarding the spontaneous power of gamma oscillations, induced power during the ASSR period (0–512 ms) was calculated by subtracting evoked power from total power, in accordance with a previous study [13, 14]. In the previous study, induced powers during baseline and ASSR periods were significantly correlated with ASSR in patients with chronic schizophrenia, whereas only induced power during the ASSR period was significantly correlated with ASSR in healthy individuals. Therefore, we measured induced power during the ASSR period because it may be a more robust measure. It may also be useful to assess the relationship of induced power during this period with ASSR. We transformed EEG data to power spectra for each trial, then calculated total power as the average power spectrum across trials. Evoked power was calculated as the power spectrum of averaged EEG data across trials. Fast Fourier transform with a 10% Hanning window was used to calculate power spectra. The frequency range for the analysis was 30–100 Hz (excluding the notch filter interval of 45–55 Hz), in accordance with a previous study [13, 14].

### Statistical analyses

For ITC analyses, a repeated-measures ANOVA was performed with time (0–100 and 300–400 ms) as the within-subjects factor and group (HC, UHR, and ROS) as the between-subjects factor. To evaluate the ITC for each time block and spontaneous power, we performed one-way ANOVAs to assess differences among the groups. To evaluate significant main effects, we performed post hoc Tukey’s honest significant difference tests. We used the Greenhouse-Geiser epsilon adjustment and set the statistical significance threshold at *P* < 0.05. Statistical analyses were performed using SPSS software (version 25; IBM Corp., Armonk, NY, USA).

Correlations between the ITC (early-latency [0–100 ms] and late-latency [300–400 ms]) and spontaneous power of gamma oscillations were evaluated in the HC, UHR, and ROS groups. Correlations between EEG measures and the PANSS subscale of hallucinatory behavior were assessed in the ROS group. Correlations between EEG measures and medication doses (equivalent diazepam and chlorpromazine doses) were assessed in the UHR and ROS groups. Pearson’s product-moment correlation coefficient (two-tailed, *P*< 0.05) was used without corrections for multiple comparisons because of the exploratory methods used for analyses.

## Results

### ASSR

**Fig. 1:**
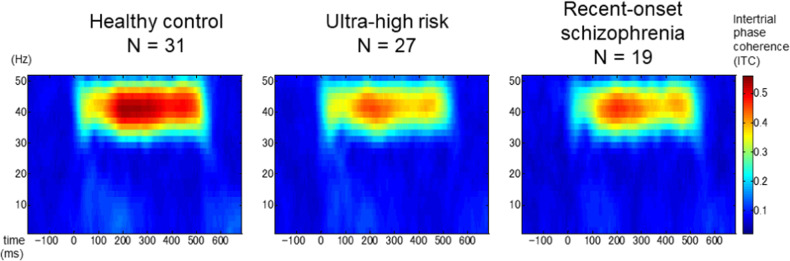
Intertrial phase coherence (ITC) in each group. Grand averages of time-frequency maps for 40-Hz ITC at the frontocentral electrode are shown for healthy controls, ultra-high-risk individuals, and patients with recent-onset schizophrenia.

In all groups, ASSR was observed in the stimuli frequency (Fig. 1). For analyses of the latency effect of ITC, a significant main effect of time (*F*[1,74] = 90.38, *P* < 0.001) and a group × time interaction (*F*[2,74] = 8.47, *P* < 0.001) were observed, consistent with findings in our previous study [10]. One-way ANOVAs for the early-latency (0–100 ms) and late-latency (300–400 ms) blocks revealed a significant main effect of group (early: *F*[2,74] = 3.62, *P* = 0.03; late: *F*[2,74] = 6.41, *P* = 0.003). In the early-latency block, the ITC was significantly lower for the ROS group than for the UHR group (*P* = 0.04). In the late-latency block, ITCs were lower for the ROS (*P* = 0.02) and UHR (*P* = 0.007) groups than for the HC group.

### *Spontaneous* power of gamma oscillations

**Fig. 2:**
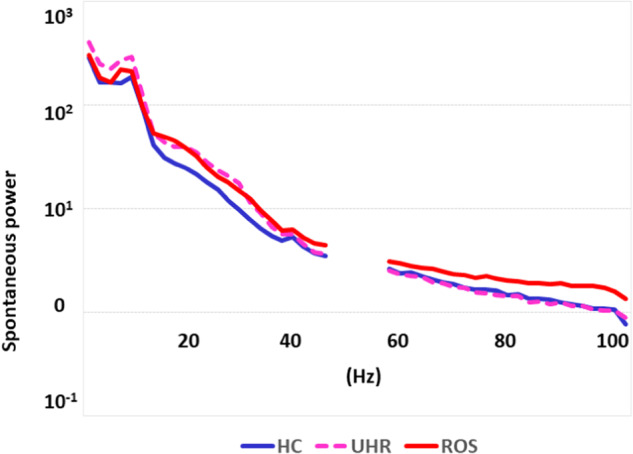
Spontaneous power spectra during the auditory steady-state response period (0–512 ms) at the frontocentral electrode in each group. Blue, dotted, and red lines indicate power spectra in healthy controls (HCs), ultra-high-risk (UHR) individuals, and patients with recent-onset schizophrenia (ROS), respectively. Because a notch filter (50 Hz) was applied, the frequencies at 45–55 Hz were not analyzed.

Figure 2 shows the power spectra of spontaneous activity during the ASSR period (0–512 ms) at FCz. One-way ANOVA showed no significant main effect of group (mean ± standard deviation, HC: 2.541 ± 0.966 vs. UHR: 2.778 ± 1.866 vs. ROS: 3.489 ± 2.562; *F*[2,74] = 1.70, *P* = 0.19). Although the spontaneous power of gamma oscillations was higher in the ROS group than in the HC and UHR groups, post hoc Tukey’s honest significant difference tests showed no significant differences in the spontaneous power of gamma oscillations among the ROS, HC, and UHR groups (mean difference for HC ± standard error: −0.237 ± 0.471, *P* = 0.87; UHR: −0.948 ± 0.521; *P* = 0.17).

### Correlations between the ASSR and spontaneous power of gamma oscillations

**Fig. 3:**
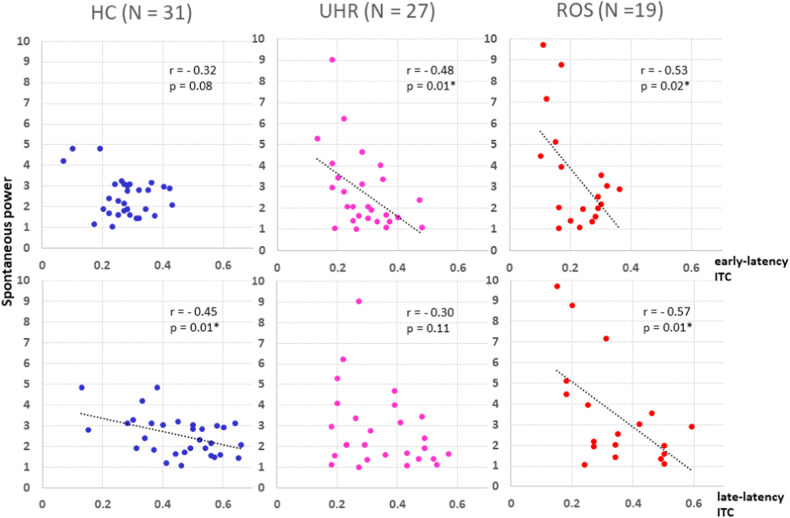
Associations between the spontaneous power of gamma oscillations and intertrial phase coherence (ITC) in healthy controls (HCs), ultra-high-risk (UHR) individuals, and patients with recent-onset schizophrenia (ROS).

Figure 3 shows the correlations between ASSR and the spontaneous power of gamma oscillations in each group. In the HC group, the spontaneous power of gamma oscillations was negatively correlated with late-latency ITC (*r* = −0.45, *P* = 0.01) but not with early-latency ITC (*r* = −0.32, *P* = 0.08). In the UHR group, the spontaneous power of gamma oscillations was correlated with early-latency ITC (*r* = −0.48, *P* = 0.01) but not with late-latency ITC (*r* = −0.30, *P* = 0.11). In the ROS group, the spontaneous power of gamma oscillations was correlated with both early- and late-latency ITC (early: *r* = −0.53, *P* = 0.02; late: *r* = −0.57, *P* = 0.01).

### Correlations with the hallucinatory behavior score and medication dosage

**Fig. 4:**
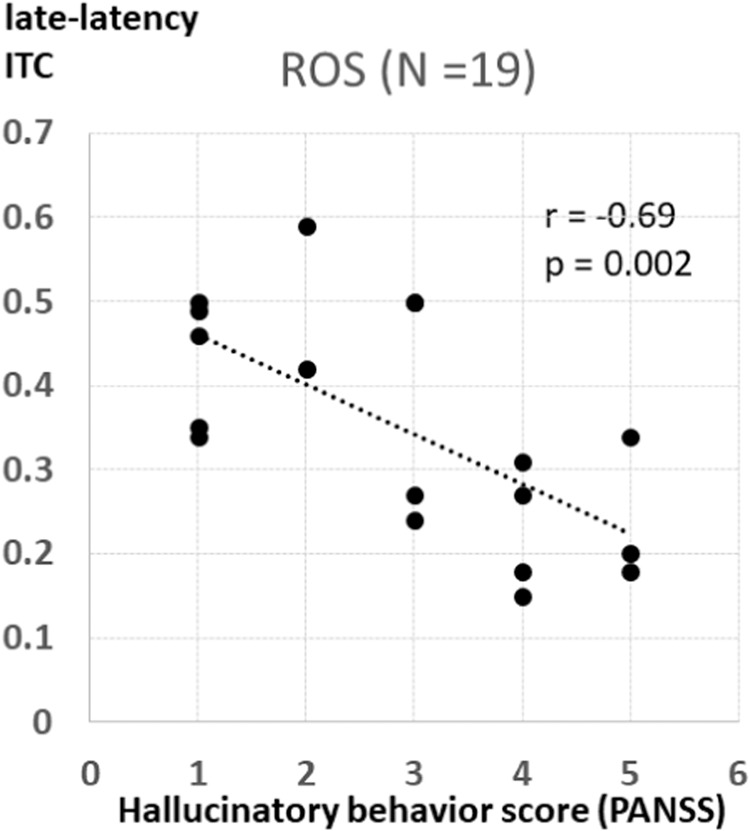
Association between gamma oscillation and hallucinatory behavior score. Association between gamma oscillation and hallucinatory behavior score. Late-latency intertrial phase coherence (ITC) was associated with the hallucinatory behavior score of the Positive and Negative Syndrome Scale (PANSS) in patients with recent-onset schizophrenia. Suppl. Head maps of the intertrial phase coherence (ITC) (top row) and spontaneous power of gamma oscillations (bottom row) in healthy controls (HCs), ultra-high-risk (UHR) individuals, and patients with recent-onset schizophrenia (ROS).

Previous studies have shown that ASSR and spontaneous power of gamma oscillations are correlated with auditory hallucinations in patients with schizophrenia [7, 13, 53]. Based on these studies, we explored the correlations of ITC and spontaneous power of gamma oscillations with hallucinations in the ROS group (Fig. 4). Using Spearman’s rank correlation coefficient, as in our previous study [10], we found that ITC was significantly correlated with the hallucinatory behavior score (P3) of the PANSS (early-latency ITC: *r* = −0.54, *P* = 0.02; late-latency ITC: *r* = −0.69, *P* = 0.002). However, the spontaneous power of gamma oscillations was not significantly correlated with the hallucinatory behavior score (*r* = 0.36, *P* = 0.14).

There were no significant correlations between EEG measures (early- and late-latency ITC and spontaneous gamma oscillations) and medication doses (antipsychotics and benzodiazepines) in the ROS group (antipsychotics: |*r* | < 0.14, *P* > 0.57; benzodiazepines: |*r*s | < 0.17, *P* > 0.50). In the UHR group, ITC was not significantly correlated with medication doses (antipsychotics: |*r* | < 0.29, *P* > 0.14; benzodiazepines: |rs | < 0.34, *P* > 0.08). However, the spontaneous power of gamma oscillations was significantly correlated with antipsychotic dose in the UHR group (*r* = 0.59, *P* = 0.001). The spontaneous power of gamma oscillations was not significantly correlated with benzodiazepine dose in the UHR group (*r* = −0.06, *P* = 0.78).

## Discussion

We found reduced gamma-band ASSR and a negative correlation between gamma-band ASSR and spontaneous power in patients with ROS. Additionally, the gamma-band ASSR was correlated with the hallucinatory behavior score of the PANSS. The correlation between the gamma-band ASSR and hallucinatory behavior score may indicate disease progression because it was identified in other studies of patients with chronic disease [13], but not in our previous study [10] of patients with early-stage (first-episode) schizophrenia. Alterations in gamma oscillations differed between the UHR and ROS groups. Although the spontaneous power of gamma oscillations was higher in the ROS group than in the HC and UHR groups, the differences were not statistically significant. To our knowledge, the present study is the first to demonstrate a correlation between the gamma-band ASSR and spontaneous power in the early stages of schizophrenia.

Consistent with findings in our previous study [10], unchanged early-latency (0–100 ms) and reduced late-latency (300–400 ms) gamma-band ASSRs were observed in the UHR group, whereas both early- and late-latency ASSRs were significantly reduced in the ROS group. Previous studies also explored the time course of ASSR [6, 8, 54, 55]. In particular, late-latency ASSRs were more affected in patients with psychosis or schizophrenia, suggesting that early- and late-latency gamma-band ASSRs reflect alterations of brain function after and before psychosis onset, respectively. The differences between early- and late-latency gamma-band ASSRs may be attributable to distinct underlying mechanisms and generators. Feature analysis of waveforms showed that ASSR is composed of early-transient and late-sustained components [52]. Our previous electrocorticography study revealed that frequency tuning characteristics differed between early- and late-latency ASSRs. In particular, the late-latency ASSR peaked at 40 Hz, whereas the frequency tuning curve for early-latency ASSR was generally flat, indicating weak frequency selectivity [51]. Recently, frequency-dependent contributions of specific GABAergic neurons have been identified in rodents [56]. Therefore, early-latency ASSR is not enhanced at any specific frequency, suggesting that the generating circuit is composed of molecules with various frequency selectivities and various cells other than parvalbumin-positive neurons. In contrast, the late-latency ASSR shows a strong peak in the gamma band, suggesting that the generating circuit is mainly composed of parvalbumin-positive neurons, a major driving circuit for gamma oscillations. Cluster analysis of time components derived from a clinical dataset may be useful for confirming our results and hypotheses in future studies.

The spontaneous power of gamma oscillations was significantly negatively correlated with early- and late-latency gamma-band ASSRs in the ROS group. These findings are consistent with the results of a previous study involving patients with chronic schizophrenia [13]. Differences in the clinical stages of patients with ROS and chronic schizophrenia may explain why the increase in spontaneous power of gamma oscillations was not statistically significant in the ROS group. These abnormal relationships between spontaneous and task-related activities in the ROS and chronic schizophrenia groups may reflect altered internally and externally oriented cognition in patients with schizophrenia [12]. Excessive changes in activity (increased or decreased compared with average) may impair normal cognition and be associated with psychopathological symptoms, such as delusions and hallucinations. Future studies should develop an index reflecting the “state dependence” described by Northoff and Gomez-Pilar [12] of gamma-band ASSR (task-related activity) and spontaneous power of gamma oscillations (pre-stimulus activity); they should also explore relationships with psychopathological symptoms.

The correlation between reduced gamma-band ASSR and increased spontaneous power of gamma oscillations in patients with ROS may reflect altered excitation/inhibition balance produced by GABAergic interneurons and pyramidal neuronal circuits [57]. GABAergic interneurons play an important role in narrow-gamma-band rhythmic activity [58], including the gamma-band ASSR. Furthermore, the spontaneous power generally exhibits a broadband high frequency, which has been correlated with higher multi-unit activities [59, 60]. The disruption of the excitation/inhibition balance in patients with ROS may be caused by NMDA receptor dysfunction because an antagonist of these receptors inhibits interneuron activities, leading to reduced pyramidal cell excitation [61]. Another NMDA receptor antagonist, ketamine, produces GABAergic interneuron dysfunction [62] and increases the spontaneous power of gamma oscillations in healthy volunteers [63, 64] and rodents [15–18]. However, because the present study did not use plasma concentrations [65] or magnetic resonance spectroscopy [27] to examine NMDA or GABA function, interpretations regarding the molecular pathogenesis of electrophysiological indices should be performed cautiously.

In contrast, reduced late-latency gamma-band ASSR was not correlated with the spontaneous power of gamma oscillations, whereas unaltered early-latency gamma-band ASSR was correlated with the spontaneous power of gamma oscillations in UHR individuals. These findings differed from the results in patients with ROS. Because the spontaneous power of gamma oscillations was correlated with the late-latency, but not early-latency, gamma-band ASSR in HCs, correlations between the gamma-band ASSR and spontaneous power of gamma oscillations differed between UHR individuals and HCs. These findings suggest the existence of specific molecular pathological processes in UHR individuals.

Our study had several limitations. First, the patients were receiving medications. Previous studies have shown that antipsychotic dose is not correlated with the spontaneous power of gamma oscillations [13, 14] or gamma-band ASSR [6, 7, 10, 66]. Similarly, we found no significant correlations between gamma-band ASSRs and medication doses (antipsychotics or benzodiazepines) in the UHR and ROS groups. However, the spontaneous power of gamma oscillations was significantly correlated with antipsychotic dose in the UHR group. Only seven individuals in the UHR group and none in the ROS group were untreated. Although it is difficult to analyze untreated individuals alone, an exploratory comparison of the spontaneous power of gamma oscillations (via *t* tests) between HCs and untreated UHR individuals did not reveal any significant differences (t7.2 = 0.04, *P* = 0.97), similar to the original analysis. Furthermore, analysis of the correlation between the spontaneous power of gamma oscillations and early-latency ITC using antipsychotic dose as a covariate did not change the results (early: *r* = −0.39, *P* = 0.048; late: *r* = −0.22, *P* = 0.284). Further research is needed to identify the potential effects of antipsychotic dose on gamma oscillations. Future studies in benzodiazepine-naïve patients are warranted to explore GABAergic interneuron dysfunction. Second, this was a cross-sectional study. Although we found a correlation between reduced gamma-band ASSRs and increased spontaneous power of gamma oscillations in the ROS group, but not the UHR group, further longitudinal studies are needed to determine whether these alterations develop before or after psychosis onset. Future studies should also examine the longitudinal changes in each group; further confirmation in large-scale studies is also warranted. In the present study, the increase in the spontaneous power of gamma oscillations did not reach the significance threshold in the ROS group. However, the analysis was underpowered, and our results should be interpreted with caution. Although we evaluated the correlation between gamma-band ASSR and hallucinatory behavior score in an exploratory manner, correlations with overall dimensions (positive, negative, and general) should also be examined. If corrections for multiple comparisons (30 items) are applied, the present correlations may not remain statistically significant. We examined correlations between ASSR and aggregate positive symptoms (P1 delusion, P3 hallucinatory behavior, P5 grandiosity, and G9 unusual thought content) based on the five-factor model [67]; we found significant correlations with late-latency ITC (*r* = −0.48, *P* = 0.04), but not early-latency ITC (*r* = −0.15, *P* = 0.57). Differences in the correlations of ASSR with hallucination alone and with aggregate positive symptoms may have occurred because hallucination is a symptom independent of other positive symptoms and has recently been proposed as a predictive coding framework in computational psychiatry [68]. Alternatively, the results may be related to the lack of correction for multiple comparisons. Finally, because we analyzed the spontaneous power of gamma oscillations during ASSR, rather than at rest or baseline (pre-stimulus), our results do not represent background power during the task [69, 70]. We analyzed the spontaneous power of gamma oscillations during ASSR because we did not collect resting-state data before the task; our objectives were to examine spontaneous power during the ASSR task, similar to previous studies of the chronic phase of schizophrenia, and to determine the effects of ASSR reduction in the early stage of the disease. Additional research is needed to explore the spontaneous power of gamma oscillation at rest.

In conclusion, we found reduced gamma-band ASSRs and identified correlations with increased spontaneous power of gamma oscillations. The pattern of alterations in gamma oscillations differed between UHR individuals and patients with ROS, suggesting that the disruption of neural dynamics between non-stimulus-locked/task modulation occurs after psychosis onset. The spontaneous power of gamma oscillations and gamma-band ASSR may provide insights into the mechanisms that underlie altered cognition and GABAergic/glutamatergic dysfunction in the early stages of schizophrenia.

## Supplementary information

Supplementary Figures
