# Supplementary figures and images for: Alterations of auditory-evoked gamma oscillations are more pronounced than alterations of spontaneous power of gamma oscillation in early stages of schizophrenia
Source: Transl Psychiatry. 2023 Jun 27;13:218. doi: 10.1038/s41398-023-02511-5 (PMC10293250; doi:10.1038/s41398-023-02511-5)

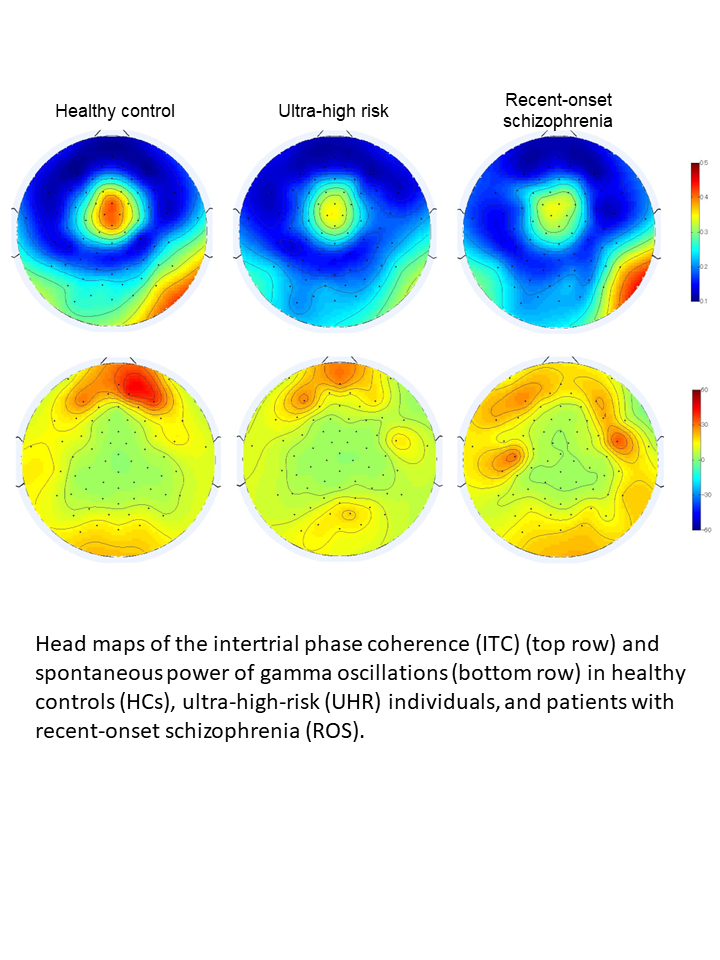

Supplement: Supplementary file 1 — Supplementary Figures [file 41398_2023_2511_MOESM1_ESM.tif]
